# Immune Molecules’ mRNA Expression in Porcine Alveolar Macrophages Co-Infected with Porcine Reproductive and Respiratory Syndrome Virus and Porcine Circovirus Type 2

**DOI:** 10.3390/v15030777

**Published:** 2023-03-17

**Authors:** Zhiying Cui, Likun Zhou, Xingxing Hu, Shijie Zhao, Pengli Xu, Wen Li, Jing Chen, Yina Zhang, Pingan Xia

**Affiliations:** 1 College of Veterinary Medicine, Henan Agricultural University, Zhengdong New District Longzi Lake 15#, Zhengzhou 450046, China; 2 College of Life Science, Henan Agricultural University, Zhengdong New District Longzi Lake 15#, Zhengzhou 450046, China; 3 Zhongnong Huada (Wuhan) Testing Technology Co., Ltd., Luoshi South Road#519, Hongshan District, Wuhan 430070, China

**Keywords:** immune molecules, immunomodulation, co-infection, PAMs, PCV2, PRRSV

## Abstract

Porcine reproductive and respiratory syndrome virus (PRRSV) and porcine circovirus 2 (PCV2) are economically important pathogens in swine, and pigs with dual infections of PCV2 and PRRSV consistently have more severe clinical symptoms and interstitial pneumonia. However, the synergistic pathogenesis mechanism induced by PRRSV and PCV2 co-infection has not yet been illuminated. Therefore, the aim of this study was to characterize the kinetic changes of immune regulatory molecules, inflammatory factors and immune checkpoint molecules in porcine alveolar macrophages (PAMs) in individuals infected or co-infected with PRRSV and/or PCV2. The experiment was divided into six groups: a negative control group (mock, no infected virus), a group infected with PCV2 alone (PCV2), a group infected with PRRSV alone (PRRSV), a PCV2–PRRSV co-infected group (PCV2–PRRSV inoculated with PCV2, followed by PRRSV 12 h later), a PRRSV–PCV2 co-infected group (PRRSV–PCV2 inoculated with PRRSV, followed by PCV2 12 h later) and a PCV2 + PRRSV co-infected group (PCV2 + PRRSV, inoculated with PCV2 and PRRSV at the same time). Then, PAM samples from the different infection groups and the mock group were collected at 6, 12, 24, 36 and 48 h post-infection (hpi) to detect the viral loads of PCV2 and PRRSV and the relative quantification of immune regulatory molecules, inflammatory factors and immune checkpoint molecules. The results indicated that PCV2 and PRRSV co-infection, regardless of the order of infection, had no effect on promoting PCV2 replication, while PRRSV and PCV2 co-infection was able to promote PRRSV replication. The immune regulatory molecules (IFN-α and IFN-γ) were significantly down-regulated, while inflammatory factors (TNF-α, IL-1β, IL-10 and TGF-β) and immune checkpoint molecules (PD-1, LAG-3, CTLA-4 and TIM-3) were significantly up-regulated in the PRRSV and PCV2 co-infection groups, especially in PAMs with PCV2 inoculation first followed by PRRSV. The dynamic changes in the aforementioned immune molecules were associated with a high viral load, immunosuppression and cell exhaustion, which may explain, at least partially, the underlying mechanism of the enhanced pulmonary lesions by dual infection with PCV2 and PRRSV in PAMs.

## 1. Introduction

Porcine reproductive and respiratory syndrome virus (PRRSV) and porcine circovirus type 2 (PCV2) are two of the most significant and important pathogens affecting the swine industry, resulting in tremendous economic losses worldwide [1,2]. PRRSV is a single-stranded RNA virus in the family *Arteriviridae*, which is the etiologic agent that causes porcine reproductive and respiratory syndrome (PRRS). Typical clinical symptoms of PRRS are characterized by severe respiratory disease in newborn/weaned piglets and growing pigs, as well as reproductive failure in pregnant pigs [3]. According to the recently updated taxonomy, PRRSV is classified into two distinct species based on its 3′-terminal structural genes: PRRSV-1 and PRRSV-2 [4]. Although these two subtypes of PRRSV cause similar clinical diseases in the infected pigs, only 55–70% of nucleotides and 50–80% of amino acids are similar in their various genes, and antigenic heterogeneity can be observed between two strains [5,6]. PCV2 is a single-stranded DNA virus in the family *Circoviridae* and is considered the causal agent of porcine circovirus-associated diseases (PCVAD) [7]. PCV2 is divided into eight genotypes, including the previously described genotypes, PCV2a to PCV2f, and the two new genotypes PCV2g and PCV2h [8]. It has been reported that concurrent infections with different PCV2 genotypes have been detected in the same pig, resulting in inter- and intra-genotype recombination [2]. However, different research groups have found that infection with PCV2 alone did not lead to obvious clinical disease and most single infections of PCV2 resulted in subclinical symptoms, with significant immunosuppression in pigs [9,10,11]. Subsequently, the immunosuppression induced by PCV2 infection is beneficial against secondary infection of other swine pathogens, including PRRSV (52%), *Mycoplasma hyopneumoniae* (36%), bacterial septicemia (14%) or pneumonia (7.6%) and swine influenza virus (SIV) (5.4%) [12]. Notably, co-infection of PRRSV and PCV2 caused more severe respiratory disease, as well as increase higher morbidity and mortality of piglets and severe pulmonary lesions [13,14,15,16].

Porcine alveolar macrophages (PAMs), which are the primary responders of the host’s defense system against pathogen invasion, are the primary target cells of PRRSV and PCV2. It is known that PRRSV and PCV2 impair the innate immunity, which leads to an inefficient adaptive immune response, inducing immunosuppression [17]. Immunosuppression induced by PRRSV and PCV2 leads to vaccine failure, immune tolerance and a long-term subclinical state in infected pigs [18]. However, neither the exact mechanisms caused by co-infection of PRRSV and PCV2 nor their pathogenesis have been elucidated yet. The presence of lymphocyte depletion and/or granulomatous lymphadenitis in PRRSV and PCV2 co-infected pigs suggests a significant role of immune-related signaling factors, inflammatory factors and immune checkpoint molecules. Immune-related signaling factors and inflammatory factors have previously been shown to be essential modulators of the immune response [19]. Immune checkpoint molecules are regulatory receptors expressed on immune cells which are able to maintain self-tolerance and modulate the breadth of the effector immune responses in peripheral tissues [20,21]. Therefore, the objective of the present study was to evaluate the kinetic changes in immune regulatory molecules, inflammatory factors and immune checkpoints in PAMs either individually infected or co-infected with PRRSV and/or PCV2, and to illuminate their correlation with the immunosuppression and synergistic pathogenesis mechanisms during concurrent infection with PRRSV and PCV2, so as to provide a theoretical basis and effective targets for the prevention and control of related diseases.

## 2. Materials and Methods

### 2.1. Viruses and Cells

The PRRSV HeN-3 strain (GenBank ID: ON645930, PRRSV-2) used in this study has been described previously [22]. The PCV2b, which was a kind gift from Qianyue Jin (Henan Academy of Agricultural Sciences, Zhengzhou, China), was used for all experiments. All animals’ tissue collection procedures were performed according to protocols approved by the Animal Care and Use Committee of Henan Agricultural University (HNND2020112613, approval date: 26 November 2020). Three six-week-old, healthy, Large White–Dutch Landrace crossbred piglets from the same litter were obtained from a herd in Henan Province. All three tested negative for both PCV2 and PRRSV. PAMs were collected by bronchoalveolar lavage as previously described [23]. Before use, the cells were confirmed as negative for PCV2, PRRSV, pseudorabies virus (PRV) and classical swine fever virus (CSFV) by PCR. PAMs were maintained in RPMI-1640 medium (Beijing Solarbio Science & Technology Co., Ltd., Beijing, China) supplemented with 2 mM L-glutamine, 1 mM sodium pyruvate, 50 U/mL penicillin, 50 µg/mL streptomycin and 10% fetal bovine serum (FBS) (Shanghai Excell Biological Technology, Shanghai, China). 

### 2.2. Trial Design

The experiment was divided into 6 groups: a negative control group (mock, no infected virus), a group infected with PCV2 alone (PCV2), a group infected with PRRSV alone (PRRSV), a PCV2–PRRSV co-infected group (PCV2–PRRSV inoculated with PCV2, followed by PRRSV 12 h later), a PRRSV–PCV2 co-infected group (PRRSV–PCV2 inoculated with PRRSV, followed by PCV2 12 h later) and a PCV2 + PRRSV co-infected group (PCV2 + PRRSV, inoculated with PCV2 and PRRSV at the same time) (Table 1). PAMs in the PCV2 alone group (PCV2), the PRRSV alone group (PRRSV) and the PCV2 and PRRSV co-infected groups (PCV2–PRRSV, PRRSV–PCV2 and PCV2 + PRRSV) were inoculated with PRRSV or/and PCV2 at a multiplicity of infection (moi) of 1. PAM samples were collected at 6, 12, 24, 36 and 48 hpi with the second virus, respectively. PAMs in the mock group were inoculated with an equal amount of RPMI-1640 medium.

### 2.3. Immunofluorescence Assay (IFA)

PAMs in the mock group and those individually infected with PRRSV or PCV2 were fixed with 4% paraformaldehyde followed by treatment with 0.1% Triton-X100 for 10 min. The PAMs were closed with 5% BSA for 2 h, then washed three times with PBST. Subsequently, the PAMs were incubated with PRRSV N protein-specific monoclonal antibody (Zoonogen, Beijing, China) or PCV2 Cap protein-specific monoclonal antibody (gifted by Qianyue Jin, Henan Academy of Agricultural Sciences) for 1 h, washed three times with PBST, incubated with fluorescein goat anti-mouse IgG(H + L) or Alexa Fluor TM 546 goat anti-mouse IgG (H + L) (Invitrogen, Waltham, MA, USA) for 1 h and then dyed for 10 min with DAPI. Finally, this was observed and these images were taken by fluorescence microscopy. 

### 2.4. Quantitative Real-Time RT-PCR Assay (RT-qPCR)

#### 2.4.1. Detection of PRRSV RNA

PRRSV RNA was extracted from cell samples using a Viral RNA Mini Kit (Qiagen, GmbH, Dusseldorf, Germany), and the RNA was reverse transcribed using a PrimeScript RT reagent kit (TaKaRa, Tokyo, Japan). PRRSV RNA was detected using the primers targeting the conserved region of ORF7 in PRRSV genomes. For the detection of PRRSV genome copy numbers, a standard curve was generated using serially diluted standard plasmids. The standard formula was y = −3.318x + 38.879, R^2^ = 0.999 (x represented the common logarithm of PRRSV copy numbers; y represented ct values). The PRRSV loads were calculated using the standard formula. Viral loads were represented as the mean logarithm of viral genome copy numbers per milliliter of serum.

#### 2.4.2. Detection of PCV2 DNA

PCV2 DNA was extracted from the cell samples using a Viral DNA Mini Kit (QIAGEN, GmbH, Dusseldorf, Germany). PCV2 DNA was detected using the primers targeting the conserved region of ORF2 in PCV2 genomes. For the detection of PCV2 genome copy numbers, a standard curve was generated using serially diluted standard plasmids. The standard formula was y = −3.9722x + 39.03, R^2^ = 0.999 (x represented the common logarithm of PCV2 copy numbers; y represented ct values). The PCV2 loads were calculated using the standard formula. Viral loads were represented as the mean logarithm of viral genome copy numbers per milliliter of serum. 

#### 2.4.3. Detection of Porcine Immune Molecules

PAMs were collected at 6 h, 12 h, 24 h, 36 h and 48 h post-infection. The total RNA from all groups was extracted using Trizol and reverse transcribed using a PrimeScript RT reagent kit (TaKaRa Shuzo, Tokyo, Japan). A relative quantification of the porcine immune molecules was carried out using the corresponding primers.

All RT-qPCR analysis was performed using a 7500 Fast real-time PCR system (Applied Biosystems, Waltham, MA, USA). The RT-qPCR reaction mixture consisted of 10 µL of 2 × SYBR Green Real time PCR Master Mix (TaKaRa, Tokyo, Japan), 2 µL of cDNA, 1 µL of each primer (0.4 µM) and 6 µL Rnase free water. The following program was used for RT-qPCR assays: polymerase activation at 95 °C for 20 s, followed by 40 cycles for melting at 95 °C for 15 s and extension at 60 °C for 30 s. Relative quantification of the expression of target genes for all groups was calculated using the 2^−ΔΔct^ method. All samples were tested in triplicate, and the RT-qPCR primers used in this study are listed in Table 2. 

### 2.5. Viral Titer Assay (TCID_50_)

In order to analyze the viral load of PRRSV or PCV2, the viral supernatants from cell cultures in all groups were collected at the indicated time points after virus inoculation. The determination of viral TCID_50_ was performed by the Reed–Muench method, as described previously [24].

### 2.6. Statistical Analysis

Data were expressed as the mean ± standard error of mean from three independent experiments. All statistical analyses were performed using the GraphPad Prism software (version 8.0) and Student’s *t*-test. A value of *p* < 0.05 was considered significant. The degree of statistical significance is indicated by asterisks (*** *p* < 0.001, ** *p* < 0.01, * *p* < 0.05).

## 3. Results

### 3.1. Confirmation of PRRSV or PCV2 Infection in PAMs

**Figure 1:**
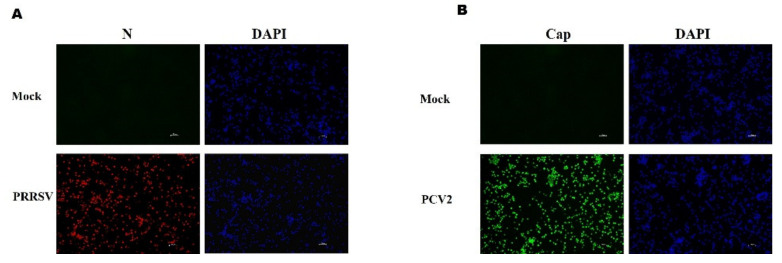
Immunofluorescence antibody staining for virus-infected PAMs by IFA. (**A**) Mock-inoculated and PRRSV-infected PAMs were stained with PRRSV N protein (red)-specific monoclonal antibody. (**B**) Mock-inoculated and PCV2-infected PAMs were stained with PCV2 Cap protein (green)-specific monoclonal antibody. Magnification: ×100.

An immunofluorescence assay (IFA) revealed red and green fluorescent cells in the infected PAMs, indicating that the PAMs were successfully infected by PRRSV (Figure 1A) and PCV2 (Figure 1B), respectively. Neither PRRSV nor PCV2 was detected in the mock-inoculated PAMs. Therefore, these data indicate that PRRSV and PCV2 successfully infected PAMs.

### 3.2. PRRSV Virologic Parameters

**Figure 2:**
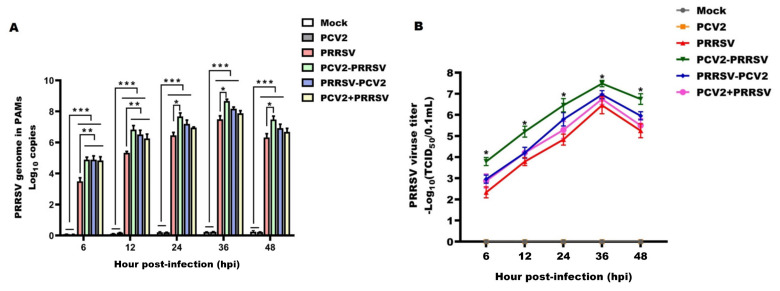
PRRSV viral load of different infection groups. PAMs in the PCV2 alone group (PCV2), the PRRSV alone group (PRRSV) and the PCV2 and PRRSV co−infected group (PCV2−PRRSV, PRRSV−PCV2 and PCV2 + PRRSV) were inoculated with PRRSV and/or PCV2 at a MOI of 1, respectively. PAMs in the mock group were inoculated with equal RPMI−1640 medium. Then, PAMs from these groups were collected at 6 h, 12 h, 24 h, 36 h and 48 h post−infection, respectively. (**A**) The levels of viral RNA were measured by RT−qPCR. (**B**) The viral titer of PRRSV in the supernatants was analyzed by TCID_50_. Data are expressed as means ± SD of three independent experiments. Statistically significant differences for the PCV2 and PRRSV co−infected groups vs. the mock−inoculated group and the PCV2 alone group are indicated (*** *p* < 0.001). Significant differences between groups are also represented (** *p* < 0.01, * *p* < 0.05).

In order to evaluate the impact of PCV2 co-infection on PRRSV parameters, we then focused on PRRSV virologic parameters. PRRSV genomic copies quickly increased in PRRSV-infected PAMs, reaching a peak at 36 hpi and then decreasing (Figure 2A, quantification; Figure 2B, TCID_50_). At 6 to 12 hpi, the PRRSV genomic copies in the PCV2–PRRSV, PRRSV–PCV2 and PCV2 + PRRSV groups were significantly higher compared with the PRRSV alone group Notably, the PRRSV genomic copies in the PCV2–PRRSV group were significantly higher compared with the PRRSV alone group throughout the period of infection, suggesting that co-infection could promote PRRSV replication. PCV2, prior to PRRSV infection (PCV2–PRRSV), had the strongest effect on PRRSV replication efficiency, while the PCV2 + PRRSV group had the weakest effect on PRRSV replication efficiency (Figure 2). PRRSV genomic copies were not detected in the mock or PCV2 alone groups at any time point. These results indicate that PCV2 and PRRSV co-infection promoted PRRSV replication.

### 3.3. PCV2 Virologic Parameters

**Figure 3:**
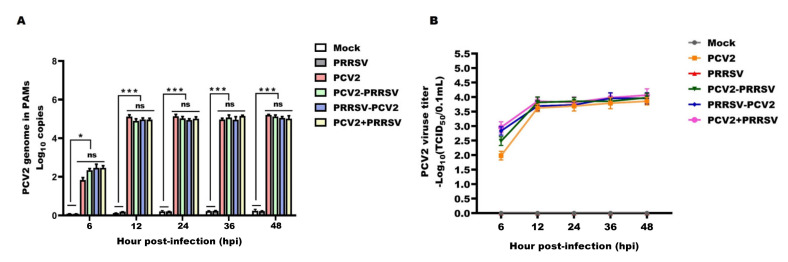
PCV2 viral load of different infection groups. PAMs in the PCV2 alone group (PCV2), the PRRSV alone group (PRRSV) and the PCV2 and PRRSV co−infected groups (PCV2−PRRSV, PRRSV−PCV2 and PCV2 + PRRSV) were inoculated with PRRSV and/or PCV2 at a MOI of 1, respectively. PAMs in the mock group were inoculated with equal RPMI−1640 medium. Then, PAMs in these groups were collected at 6 h, 12 h, 24 h, 36 h and 48 h post−infection, respectively. (**A**) The levels of viral DNA were measured by RT−qPCR. (**B**) The viral titers of PCV2 in the cell culture supernatants were analyzed by TCID_50_. Data are expressed as means ± SD of three independent experiments. Statistically significant differences for the PCV2 and PRRSV co−infected groups vs. the mock−inoculated group and the PRRSV alone group (* *p* < 0.05, *** *p* < 0.001). No significant differences were observed for the PCV2 alone group vs. the PCV2 and PRRSV co−infected groups (ns).

After having explored the effect of PCV2 on PRRSV parameters, we then evaluated the impact of PCV2 and PRRSV co-infection on PCV2 virologic parameters. PCV2 genomic copies remained between 10^4^ copies/µL~10^5^ copies/µL from 12 to 48 hpi in the PCV2 alone group and the dual inoculation of PRRSV and PCV2 groups. However, the groups dually inoculated with PCV2 and PRRSV (PCV2–PRRSV, PRRSV–PCV2 and PCV2 + PRRSV) showed no significant differences, regardless of the infection order, compared with infection by the PCV2 alone group throughout the experimental period (Figure 3A, quantification; Figure 3B, TCID_50_). These results demonstrate that PCV2 and PRRSV co-infection had no effect on PCV2 replication. Additional studies are necessary to elucidate the mechanism(s) leading to this finding. Meanwhile, PCV2 genomic copies were not detected in the mock or PRRSV alone groups at any time point.

### 3.4. Detection of mRNA Levels of IFN-α and IFN-γ

**Figure 4:**
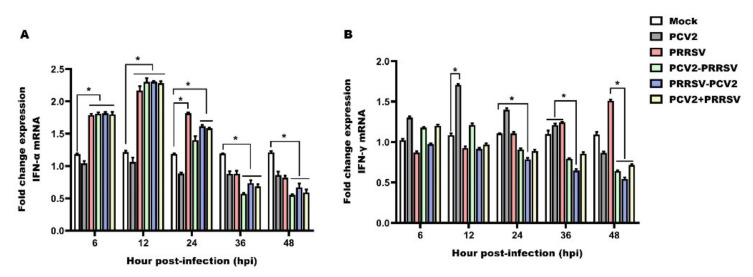
Relative mRNA expression of IFN-α and IFN-γ. PAMs in the PCV2 alone group (PCV2), the PRRSV alone group (PRRSV) and the PCV2 and PRRSV co-infected groups (PCV2–PRRSV, PRRSV–PCV2 and PCV2 + PRRSV) were inoculated with PRRSV or/and PCV2 at a MOI of 1, respectively. PAMs in the mock group were inoculated with equal RPMI-1640 medium. Then, PAMs from these groups were collected at 6 h, 12 h, 24 h, 36 h and 48 h post-infection, respectively. RT-qPCR analysis of IFN-α (**A**) and IFN-γ (**B**) mRNAs in PAMs from different groups. Data are expressed as means ± SD of three independent experiments. Significant differences between groups are represented (* *p* < 0.05).

IFN-α is considered to play a major role in antiviral defense. Thus, we addressed whether the expression of IFN-α could be modulated in the PAMs of the PCV2 and PRRSV co-infected groups and the groups infected with PCV2 or PRRSV alone. A considerable increase in the expression levels of IFN-α was observed at 6 to 12 hpi in the PRRSV alone group and in the PCV2 and PRRSV co-infected groups, showing significant differences with respect to the mock and PCV2 alone group (Figure 4A). Then, a progressive decrease until the end of the study was observed for the mRNA levels of IFN-α. For the PCV2 and PRRSV co-infected groups, the kinetics of IFN-α were similar to those observed in the PRRSV alone group, with a peak in expression at 12 hpi and a progressive decrease until 48 hpi, and always with lower values than those observed in the groups infected with PCV2 or PRRSV alone (Figure 4A). Infection with PCV2 prior to PRRSV (PCV2–PRRSV) showed a more significant inhibitory effect on IFN-α expression compared with PRRSV infection prior to PCV2 (PRRSV–PCV2) (Figure 4A). During the entire study, the expression of IFN-α in PCV2 alone group was consistently lower than in the mock group (Figure 4A). To summarize, IFN-α was more obviously down-regulated in the PCV2–PRRSV group than in PRRSV-associated infection groups at later stages (36 to 48 hpi).

IFN-γ has potent antiviral properties, and is an important mediator of cellular responses. The expression of IFN-γ was detected in PCV2 and PRRSV co-infected PAMs as well as in those infected with PRRSV or PCV2 alone. IFN-γ mRNA was found to be slightly down-regulated in the early stage (6 to 12 hpi) in the PRRSV alone group compared to the mock group; however, the IFN-γ expression level was increased in the PRRSV alone group in the late stage (24 to 48 hpi) (Figure 4B). On the contrary, the expression levels of IFN-γ were observed to increase at 6 to 12 hpi in the PCV2 alone group compared to the mock group, and to be decreased at 24 to 48 hpi in the PCV2 alone group (Figure 4B). For the PCV2 and PRRSV co-infected group, the kinetics of IFN-γ always showed lower values than those observed in the PCV2 or PRRSV alone groups at the later stages (24 to 48 hpi) (Figure 4B). Particularly, the IFN-γ expression in the PRRSV–PCV2 group was significantly lower than in the PCV2–PRRSV group and the PRRSV + PCV2 group (Figure 4B). These results suggest that IFN-γ was up-regulated at the early stage (6 to 12 hpi) and down-regulated at the late stage (36 to 48 hpi) in the PCV2-associated infection groups. The down-regulation effect of the co-infection groups was stronger than that of the PCV2 alone group, and the down-regulation effect of the PRRSV–PCV2 group was the most obvious.

### 3.5. Detection of mRNA Levels of TNF-α and IL-1β

**Figure 5:**
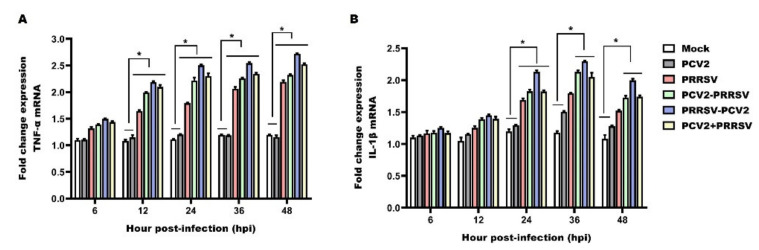
Relative mRNA expression of TNF-α and IL-1β. PAMs in the PCV2 alone group (PCV2), the PRRSV alone group (PRRSV) and the PCV2 and PRRSV co-infected groups (PCV2–PRRSV, PRRSV–PCV2 and PCV2 + PRRSV) were inoculated with PRRSV or/and PCV2 at a MOI of 1, respectively. PAMs in the mock group were inoculated with equal RPMI-1640 medium. Then, PAMs from these groups were collected at 6 h, 12 h, 24 h, 36 h and 48 h post-infection, respectively. RT-qPCR analysis of TNF-α (**A**) and IL-1β (**B**) mRNAs in PAMs with different groups. Data are expressed as means ± SD of three independent experiments. Significant differences between groups are represented (* *p* < 0.05).

TNF-α and IL-1β are acute-phase pro-inflammatory mediators that promote inflammation and induce fever and tissue injury. All PCV2 and PRRSV co-infected groups, as well as the PRRSV alone group, showed a progressive increase in the production of TNF-α compared with the mock group in PAMs during the experimental period (Figure 5A). The expression levels of TNF-α in all PCV2 and PRRSV co-infection groups were significantly up-regulated compared to the PCV2 alone group at 12 to 48 hpi (Figure 5A). Furthermore, the group infected with PRRSV prior to PCV2 (PRRSV–PCV2) showed a significant increase in the production of TNF-α than the PCV2–PRRSV group and the PCV2 + PRRSV group (Figure 5A). The PCV2 alone group showed no significant differences compared with the mock group throughout the whole study (Figure 5A). These data demonstrate that both co-infection and infection with PRRSV alone could possibly up-regulate the TNF-α mRNA level. The mRNA level of TNF-α was the highest in the PRRSV–PCV2 group.

For the PCV2 and PRRSV co-infected groups, the kinetic changes of IL-1β were similar to those observed in the PRRSV alone group, with a peak of expression at 36 hpi and a decrease at 48 hpi, and always with higher values than those observed in the PCV2 alone group and the mock group (Figure 5B). Moreover, the group infected with PRRSV prior to PCV2 (PRRSV–PCV2) showed a significant increase in the production of IL-1β compared with the PCV2–PRRSV group and the PCV2 + PRRSV group (Figure 5B). The PCV2 alone group also showed no significant differences compared to the mock group during the experimental period (Figure 5B). The results demonstrate that the expression of IL-1β reached a peak at 36 hpi in the PRRSV-associated infection groups, and the expression of IL-1 β was most obviously up-regulated in the PRRSV–PCV2 group.

### 3.6. Detection of mRNA Levels of IL-10 and TGF-β

**Figure 6:**
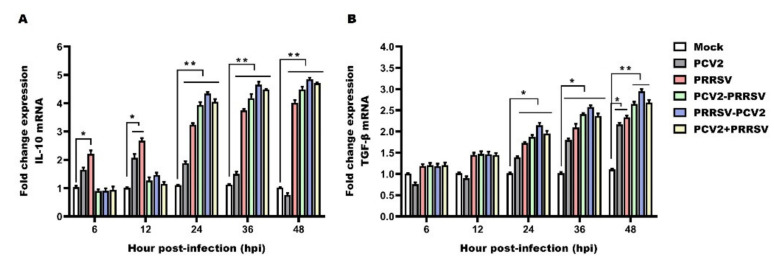
Relative mRNA expression of IL-10 and TGF-β. PAMs in the PCV2 alone group (PCV2), the PRRSV alone group (PRRSV) and the PCV2 and PRRSV co-infected groups (PCV2–PRRSV, PRRSV–PCV2 and PCV2 + PRRSV) were inoculated with PRRSV and/or PCV2 at a MOI of 1, respectively. PAMs in the mock group were inoculated with equal RPMI-1640 medium. Then, PAMs in these groups were collected at 6 h, 12 h, 24 h, 36 h and 48 h post-infection, respectively. RT-qPCR analysis of TNF-α (**A**) and IL-1β (**B**) mRNA in PAMs from different groups. Data are expressed as means ± SD of three independent experiments. Significant differences between groups are represented (** *p* < 0.01, * *p* < 0.05).

IL-10 is a well-established immunosuppressive cytokine that has been associated with hindered viral clearance. The mRNAs of IL-10 were up-regulated at the early stage (6 to 12 hpi), while the IL-10 mRNA was decreased at the late stage (24 to 48 hpi) in the PCV2 alone group compared to the Mock group (Figure 6A). The mRNA levels of IL-10 in the PRRSV alone group progressive increased compared to those in the mock group throughout the study (Figure 6A). For the PCV2 and PRRSV co-infected groups, a considerable decrease in the expression level of IL-10 was observed at 6 to 12 hpi, while significant increase at 24 to 48 hpi was found compared to the PCV2 alone group and the mock group (Figure 6A). In addition, the mRNA level of IL-10 was at its highest in the PRRSV–PCV2 group (Figure 6A). The expression of IL-10 was up-regulated at the late stage (24 to 48 hpi) in PRRSV-associated infection groups, of which the expression of IL-10 was the highest in PRRSV–PCV2 group.

Compared to the mock group, the mRNA levels of TGF-β in the PCV2 alone group was down-regulated at 6 to 12 hpi, but then a progressive increase occurred at 24 to 48 hpi (Figure 6B). The kinetic changes in TGF-β were found to progressively increase compared to the mock group and the PRRSV alone group during the experimental period (Figure 6B). At 6 to 12 hpi, the expression levels of TGF-β showed no statistically significant differences between the PCV2 and PRRSV co-infected groups and the PRRSV alone group (Figure 6B). Then, at 24 to 48 hpi, the TGF-β mRNAs in the PCV2 and PRRSV co-infected groups were significantly up-regulated compared to the PCV2 group and the mock group (Figure 6B). Furthermore, the mRNA level of TGF-β was the highest in the PRRSV–PCV2 group (Figure 6B). In conclusion, increased mRNA levels of TGF-β were observed in all infection groups at the late stage (24 to 48 hpi), and the TGF-β mRNA level showed a significant increase in the PRRSV–PCV2 group compared with the other groups.

### 3.7. Detection of mRNA Levels of Immune Checkpoints

**Figure 7:**
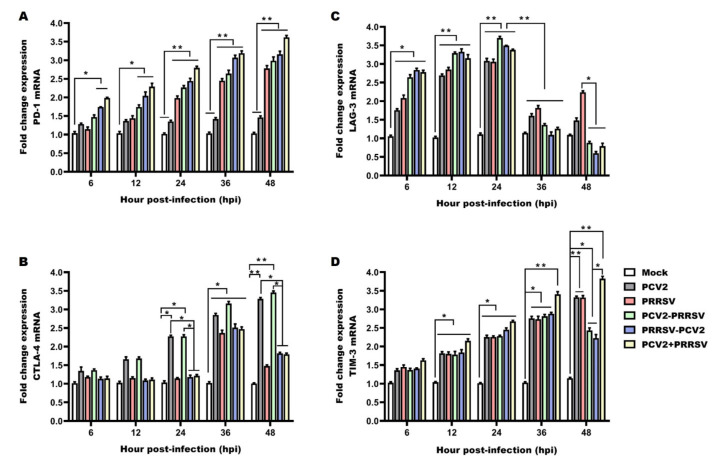
Relative mRNA expression of immune checkpoints. PAMs in the PCV2 alone group (PCV2), the PRRSV alone group (PRRSV) and the PCV2 and PRRSV co-infected groups (PCV2–PRRSV, PRRSV–PCV2 and PCV2 + PRRSV) were inoculated with PRRSV and/or PCV2 at a MOI of 1, respectively. PAMs in the mock group were inoculated with equal RPMI-1640 medium. Then, PAMs from these groups were collected at 6 h, 12 h, 24 h, 36 h and 48 h post-infection, respectively. RT-qPCR analysis of PD-1 (**A**), CTLA4 (**B**), LAG3 (**C**) and TIM3 (**D**) mRNAs in PAMs from different groups. Data are expressed as means ± SD of three independent experiments. Significant differences between groups are represented (** *p* < 0.01, * *p* < 0.05).

A progressive up-regulation of PD-1 was observed in the PRRSV alone group and the PRRSV and PCV2 co-infected groups in comparison with the mock group over the course of the study. Statistically significant differences were observed at 24 to 48 hpi with respect to the PCV2 alone group and the mock group (Figure 7A). The largest PD-1 expression increase occurred in the PCV2+ PRRSV co-infected group. The PCV2 group showed no statistical differences with the mock group (Figure 7A). These results demonstrated that the mRNA levels of PD-1 were significantly up-regulated in the PRRSV-associated infection groups, especially in the PCV2 + PRRSV co-infected group.

A remarkable up-regulation in the expression of CTLA-4 was observed in the PCV2 alone group and the PCV2–PRRSV group during the experimental period, showing significant differences with the other groups at 12 to 48 hpi (Figure 7B). At 6 to 24 hpi, the expression of CTLA-4 in the PRRSV alone group, PRRSV–PCV2 group and PCV2 + PRRSV group was not significantly different from that in the mock group (Figure 7B). At 36 hpi, the expression of CTLA-4 drastically increased, but dropped slightly at 48 hpi in the PRRSV alone group, PRRSV–PCV2 group and PCV2 + PRRSV group, although it was still significantly up-regulated with respect to the mock group (Figure 7B). In general, the expression of CTLA-4 in the PCV2 alone group and the PCV2–PRRSV infected group increased with the time since infection, while the expression of CTLA-4 in the PRRSV alone group, the PRRSV–PCV2 group and the PCV2 + PRRSV group reached a peak at 36 hpi.

The mRNAs of LAG-3 were up-regulated at 6 to 24 hpi, while the LAG3 mRNAs were decreased at 36 to 48 hpi in the PCV2 and PRRSV alone groups, but still significantly up-regulated with respect to the mock group (Figure 7C). An increase in the expression of LAG-3 in PCV2 and PRRSV co-infected PAMs from 6 to 24 hpi was detected, showing statistically significant differences compared to the mock group (Figure 7C). Later on, a considerable decrease in its expression was observed at 36 to 48 hpi, showing statistically significant differences at 48 hpi with respect to the mock group (Figure 7C). The PCV2 and PRRSV co-infected groups showed significant decreases in the production of LAG-3 compared with the PRRSV alone group at 48 hpi (Figure 7C). It can be concluded that the mRNA level of LAG-3 reached its peak at 24 hpi in all infected groups, and the mRNA level of LAG-3 in the PRRSV alone group was higher than in the co-infected groups at 48 hpi. A progressive up-regulation of TIM-3 was observed in PAMs individually infected or co-infected with PRRSV and/or PCV2 in comparison with the mock group throughout the whole study, showing statistically significant differences at 12 to 48 hpi with respect to the mock group (Figure 7D). At 36 to 48 hpi, the PCV2 + PRRSV group showed a significant increase in the production of TIM-3 compared with the other groups (Figure 7D). At 48 hpi, the expression of TIM-3 was mildly decreasing in the PCV2–PRRSV and PRRSV–PCV2 groups, but still significantly decreased in comparison with the mock group (Figure 7D). The mRNA level of TIM-3 was up-regulated in all infected groups compared with the mock group, and the mRNA level of TIM-3 in the PCV2 + PRRSV group was the highest of all the groups.

## 4. Discussion

A high prevalence of PCV2 and PRRSV co-infection is frequently observed in the global swine industry, resulting in tremendous economic losses. However, infection with PCV2 or PRRSV alone caused only subclinical symptoms, indicating that there may be a synergistic relationship between PRRSV and PCV2 in viral infection. PAMs are the primary responders of the pulmonary innate immune system; they resist against various pathogens [25,26] and are also major target cells for infection by both PCV2 and PRRSV. The present study was conducted to analyze the dynamic changes in virulence and immune molecule (IFN-α, IFN-γ, TNF-α, IL-1β, IL-10, TGF-β, PD-1, LAG-3, CTLA-4 and TIM-3) expression in PAMs either individually infected with PCV2 or PRRSV, co-infected with PCV2 and PRRSV in different orders, or simultaneously co-infected with PCV2 and PRRSV in vitro, respectively (Table 1). We hoped to gain insights into the underlying synergistic pathogenesis mechanism of PCV2 and PRRSV co-infection in PAMs to explore novel targets for improved preventative and therapeutic strategies.

Dual infection of PCV2 and PRRSV has frequently been encountered in pig herds globally. Several research groups have demonstrated that PRRSV could cause enhanced PCV2 replication, as evidenced by higher serum and tissue PCV2 loads, leading to the development of PCVAD in vivo [27,28,29,30]. Another study demonstrated that PCV2 and PRRSV co-infection influences the infection dynamics of PCV2a and PCV2b by extending PCV2 viremia and shedding in vivo [31]. In addition, some research has reported that inoculation with a modified live PRRSV vaccine followed by co-infection with PRRSV and PCV2 enhanced PCV2 replication and pathogenesis [18]. The commonality of target cell type and/or subsequent immune dysfunction may be an important factor in the enhanced replication of PCV2 following dual infection with PRRSV [27]. In this study, the virulence of PCV2 did not show an increasing trend with the advancement of infection time in the PCV2 alone group nor the PCV2 and PRRSV co-infected groups. Moreover, the proliferation of PCV2 was very low in all groups infected with PCV2, suggesting that PCV2 cannot be proliferated in PAMs. In relation to the findings of our studies, PCV2 cannot efficiently replicate in swine alveolar macrophages (AMs) unless being activated by, for example, lipopolysaccharides in vitro [32]. It has been speculated that PAMs have lost their differentiation ability in vitro, and it is also may be that PCV2 exploits this mechanism to evade the host immune response in PAMs without being recognized and degraded, leading to persistent infection. A previous study indicated that PCV2-induced IFN-α released in the supernatant of PCV2-inoculated AMs could interfere with PCV2 itself in order to infect and, subsequently, to replicate in PK-15 cells. The interference with PCV2 replication by PCV2-induced IFN-α may explain the restriction of PCV2 in the cytoplasm of monocyte/macrophage lineage cells, with no further active replication when PCV2 was inoculated alone [32]. In addition to the above factors, the different effects of PCV2 and PRRSV co-infection on PCV2 replication related to the use of different strains of PCV2 may be due to the breed of the pigs, type of inoculum (homogenate versus virus isolated in cell culture), inoculation route, dose of virus, number of passages of the virus in the cell culture, source of the pigs (conventional, gnotobiotic, colostrum-deprived or cesarean-derived pigs) and age of the inoculated pigs.

In a PCV2 and PRRSV co-infection study, it was suggested that PCV2 reduced PRRSV replication [33]. Contrarily, another study reported that piglets co-infected with PCV2 and PRRSV showed enhanced PRRSV replications, and that more severe clinical signs and lesions were observed [16]. However, it has been shown previously that PCV2 does not affect PRRSV replication or lesions [34]. It has been reported that PCV2 infection increases the rate of amino acid mutations of PRRSV during serial passages in pigs, accelerating the proliferation of PRRSV [35]. The results of this study demonstrated that the virulence of PRRSV rapidly proliferated in the PRRSV alone group, as well as in the PCV2 and PRRSV co-infected groups, in PAMs. Furthermore, the virulence of PRRSV in the PCV2 and PRRSV co-infected groups increased more significantly than in the PRRSV alone group, suggesting that co-infection could promote PRRSV replication. Infection with PCV2 prior to PRRSV had the strongest effect on PRRSV replication, indicating that PCV2 causes enhanced replication of PRRSV in PAMs. The results of the co-infection study with PCV2 and PRRSV indicate that the effect of PCV2 on the virulence of PRRSV is controversial. These differences may be correlated with the use different virus strains or with the states of the experimental animals or the cells. Therefore, future studies should be carried out in order to elucidate the mechanism(s) involved in synergistic effects during PCV2 and PRRSV co-infection.

Type I interferons IFN-α and IFN-β are best known for their ability not only to induce an antiviral state, but also to have numerous additional functions that influence the innate and adaptive immune response. Recently, it has been reported that IFN-α suppressed the immune response in some acute viral infections, which could affect the development of immunosuppression or immunopathology [36]. Our present study demonstrated that a significant amount of IFN-α mRNA was induced at the early stage (6 to 12 hpi), but a persistent reduction in IFN-α mRNA was seen at the late stage (36 to 48 hpi), in the PRRSV alone group as well as in the PCV2 and PRRSV co-infection groups. Moreover, no significant change was seen in the PCV2 alone group throughout the experimental period. These findings indicate that co-infection or PRRSV alone could induce IFN-α in PAMs at the early stage, while it could interfere with or hinder the production of IFN-α in PAMs at the late stage. Thus, it is reasonable to assume that IFN-α, produced after the early stage of viral infection, could affect the immune response of monocyte-derived macrophages, and a down-regulation in expression at the late stage may indicate a virus-modulated mechanism to enhance infection and replication. On the contrary, a research group investigated the levels of IFN-α mRNA in the PRRSV alone group and found them to be low throughout the experimental period in AMs, while they were increased in all PCV2-inoculated groups and were consistently and significantly higher than those in the PRRSV alone group [37]. The study conducted by Albina et al. showed that pre-infection of AMs with PRRSV 6 h prior to the inoculation of swine transmissible gastroenteritis virus (TGEV) in vitro resulted in a complete inhibition of TGEV-induced IFN-α production [38]. The aforementioned results indicate that the interactions among different viruses are complicated and may lead to different disease courses in the field. IFN-γ is a Th1-specific cytokine produced by macrophages, NK cells and other cell types during viral infection [39]. IFN-γ has potent antiviral properties that contribute to the control of acute viral infections, and it is an important mediator of cellular responses. The expression of IFN-γ from peripheral blood mononuclear cells (PBMCs) has been reported to significantly increase in the PCV2-infected and PRRSV/PCV2-co-infected piglets [40]. Another study showed that co-infection with PCV2 and the pseudorabies virus (PRV) promotes IFN-γ expression, suggesting that IFN-γ could protect against PCV2 and/or PRV infections [2]. During this study, it was found that IFN-γ mRNA levels were slightly up-regulated at the early stage (6 hpi) of infection with PCV2 alone and co-infection with PCV2 and PRRSV in PAMs. At the late stage (36 to 48 hpi) of infection of PAMs with PCV2 alone and co-infection with PCV2 and PRRSV, however, the IFN-γ expression level was decreased. This repressed expression may be indicative of suppressed Th1 responses facilitating viral persistence and delaying viral clearance. The increasing expression of IFN-γ mRNA in PAMs infected with PRRSV alone suggests that such an up-regulation in expression may indicate a virus-modulated mechanism to enhance infection and replication in the group infected with PRRSV alone. 

TNF-α is a pleiotropic cytokine that can induce and regulate the inflammatory response, including the release of inflammatory mediators and chemokines [41]. IL-1β is an inflammatory marker and plays a significant role in inflammation-related diseases, inducing various pro-inflammatory mediators, such as cytokines and chemokines [42]. A previous study showed that co-infection of PAMs with PRRSV and *Mycoplasma hyopneumoniae* considerably increased the expression of pro-inflammatory cytokines TNF-α and IL-1β [43]. Interestingly, as shown in the current study, PAMs co-infected with PCV2 and PRRSV and with PRRSV alone showed increased expression levels of TNF-α and IL-1β in comparison with the mock and PCV2 alone groups throughout the experimental period. Moreover, it has been demonstrated that production of TNF-α and IL-1β may induce fever and respiratory distress in PRRSV- or PCV2-infected pigs [40]. Therefore, the aforementioned results indicate that significantly increased TNF-α and IL-1β expression might be responsible for the increased inflammatory response and disease severity associated with PCV2 and PRRSV co-infection. Other research has reported that PRRSV has the ability to suppress TNF-α production at the post-transcriptional level [44]. Several studies have shown that PAMs or peripheral blood mononuclear cells (PBMCs) infected with PCV2 produce higher levels of TNF-α and IL-1β [39,45,46]. Conversely, we found that the expression of TNF-α did not significantly change in PAMs infected with PCV2 alone. This discrepancy in TNF-α production with PCV2 may be explained by the complexity of the interactions between pathogens and the immune system.

IL-10-mediated immunosuppression may play an important role in persistent viral infections. TGF-β is a crucial enforcer of immune tolerance, inhibiting the expansion and function of many components of the immune system [47,48]. In practice, several studies have shown that the level of IL-10 mRNA was increased in PAMs co-infected with both pathogens [49,50]. Consistently, we also found that IL-10 expression was significantly up-regulated by the PRRSV alone and PCV2 and PRRSV co-infection groups, and a stronger effect was observed in PRRSV–PCV2 group. As is consistent with our results, it has been shown that IL-10 mRNA levels were significantly up-regulated in the PRRSV- and PCV2-infected pigs [46,51]. Contrary to that, IL-10 expression levels in the PCV2 and PRRSV co-infected piglets were significantly lower than those in the piglets infected with PRRSV alone [40], suggesting that other mediators may be responsible for the immunosuppression in the piglets co-infected with two viruses. In addition, induction of IL-10 production could be one of the strategies exploited by PRRSV to modulate the host’s immune responses, thereby contributing to opportunistic or secondary infections and the development of PMWS in piglets [52]. Similarly, the increasing production of TGF-β was observed in PAMs with dual inoculation of PRRSV and PCV2 regardless of the order of infection, whereas a stronger effect was found in the PRRSV–PCV2 group. Therefore, the aforementioned studies’ results provide direct evidence that IL-10 and TGF-β induction by PCV2 and PRRSV co-infection impair the immune defense capability of porcine alveolar macrophages, leading to immune disorders.

Previous studies on the effects of co-infection of PRRSV and PCV2 on pigs had different findings. Jinyong Zhang et al., found that the serum concentrations of IFN-γ in pigs co-infected with PRRSV and PCV2 were significantly higher than those in the control group [53]. Peihu Fan’ team discovered that the HP-PRRSV/PCV2 co-infection group had up-regulated serum concentrations of TNF-α and IL-10, while IFN-γ was lower in the co-infection groups, especially the HP-PRRSV/PCV2 group, than in the single-infection groups [16]. Other research isolated PBMCs from infected piglets and found that the IL-10 mRNA level was lower and the TNF-α mRNA level was higher in the co-infected group than that in the PRRSV alone group [40]. Another in vitro experiment found that the expression of TNF-α in the culture supernatant of PAM cells was higher in the PRRSV–PCV2 co-infected group than in the other groups [37]. As is consistent with these studies, our study also found that co-infection promoted TNF-α expression. Moreover, the results of our study on IL-10 and IFN-γ were consistent with the findings of Peihu Fan et al., but contrary to the findings of Jinyong Zhang et al., and Fan, P et al., We speculate that animal breed, sample source, duration of infection, differences in strain, animal status and infection procedure are the main reasons for the differences in animal data in vitro and in vivo.

Immune checkpoints have emerged as a strategy executed by viruses to subvert the immune response and escape from the host’s immune defense, and its increased expression is associated with disease progression [20]. In this study, porcine alveolar macrophages were infected with different combinations of PCV2 and PRRSV and evaluated for their expression levels of selected immune checkpoints (PD-1, CTLA-4, TIM-3 and LAG-3). PD-1 and its ligand, programmed cell death ligand (PD-L1), have been the most studied immune checkpoints due to their role in modulating disease through immune suppression [54]. Increased expression of PD-1 and PD-L1 has been suggested in pigs suffering from post-weaning multisystemic wasting syndrome (PMWS) [49]. The same authors found that up-regulation of the PD-1/PD-L1 axis was associated with apoptosis in monocyte-derived DCs of both the PCV-2 single infection and PCV-2 and PRRSV-2 co-infection models. Other research has shown up-regulation of PD-1 to be associated with impairment of the immune response during acute classical swine fever virus (CSFV) infection [55]. It has previously been shown that increased PD-1 expression induces T-cell apoptosis and hinders viral clearance in adenovirus and hepatitis B virus (HBV) infections [56]. A study has demonstrated that increased PD-L1 expression in monocyte-derived dendritic cells may be an underlying mechanism of immune suppression in the development of PCVAD during PCV2 infection [57]. In the present study, we found that the mRNA levels of PD-1 were significantly up-regulated in PAMs infected with PRRSV alone, as well as those co-infected with PCV2 and PRRSV, and especially in those simultaneously infected with PCV2 and PRRSV, showing a time-dependent increase in PD-1 expression. Based on the results, we speculate that increased PD-1/PD-L1 expression may contribute to the mechanism(s) of immune suppression and apoptosis, particularly of T-cells, in cases of PCVAD. Several research groups have investigated how CTLA-4 binds with its ligand, CD80/CD86, interfering with T-cell development, proliferation and survival and suppressing negative selection at the thymus [58,59]. A previous study reported that CTLA-4 was significantly up-regulated in the thymus of PRRSV-infected piglets, and this effect was strain dependent [60]. In the current report, similar PCV2-induced CTLA-4 effects were observed in the PCV2–PRRSV group throughout the experimental period. A significant amount of CTLA-4 was also induced in the PRRSV–PCV2 and PCV2 + PRRSV groups at 36 hpi; however, the CTLA-4 expression level was decreased in the PRRSV–PCV2 and PCV2 + PRRSV groups in comparison with the PCV2 alone group and the PCV2–PRRSV group at 48 hpi. Thus, it is suggested that PCV2 single infection and PCV2 prior to PRRSV infection could increase the CTLA-4 expression. Additional studies should be conducted to determine whether this finding could be linked with the different orders of co-infection.

Previous reports have indicated that the expression levels of TIM-3 and LAG-3 were transiently up-regulated in activated CD4+ and CD8+ T-cells, exhausted CD8 T-cells, Tregs, type 1 regulatory T cells and NK cells, as well as in DCs in the case of TIM-3 [61]. This up-regulation was associated with disease progression, high viral load and cell death. Co-expression of these molecules can affect T-cell development, maturation and selection, negatively regulating the host immune response [60]. However, the expression of TIM-3 and LAG-3 in PAMs of viral infection has scarcely been studied. In our study, a progressive increase in the expression of LAG-3 was observed at the early stage (6 to 24 hpi) of PCV2 or PRRSV single infection and co-infection with PCV2 and PRRSV in PAMs. However, at the late stage (48 hpi) of co-infection with PCV2 and PRRSV in PAMs, the LAG-3 expression level was decreased, but this did not occur after infection with PCV2 or PRRSV alone. An up-regulation of TIM-3 was visible in PAMs infected with either PCV2 or PRRSV alone, as well as in those co-infected with PCV2 and PRRSV, in our study, but this effect was significantly greater in the PCV2 + PRRSV group. A previous study with peripheral blood mononuclear cells from PCV2-infected pigs was not able to identify changes in the expression of LAG-3, suggesting a low level of participation of this marker during PMWS [62]. Other reports have investigated how TIM-3 negatively regulates Th1 CD4+ and CD8+ T-cells by inducing cell death upon interaction with its ligand, galectin-9 [63]. A recent study showed that LAG-3 and TIM-3 were significantly more highly expressed in thymocytes and macrophage-like cells from PRRSV-infected piglets [60]. Collectively, the up-regulation of both LAG-3 and TIM-3 genes within virus-infected PAMs reflects they may be involved in the regulation of immune response during PCV2 and PRRSV co-infection. Future study should be explored how LAG-3 and TIM-3 act to modulate immunity in viral infections.

## 5. Conclusions

To summarize, PCV2 and PRRSV co-infection produced significant alterations in the mRNA expression of immune molecules (IFN-α, IFN-γ, TNF-α, IL-1β, IL-10, TGF-β, PD-1, LAG3, CTLA4 and TIM3) in PAMs, which may trigger immunosuppression and, thus hinder viral clearance, lead to persistent infection and increase disease severity. The differential transcription expressions of immune molecules which we observed may provide insights into the underlying immunological response that occurs in PAMs following PCV2 and PRRSV co-infection. Continued research should be conducted to shed light on the exact impact of the dynamic changes in immune molecules involved in PAMs after PCV2 and PRRSV co-infection. This information could ultimately help researchers to better understand the synergistic pathogenesis mechanism of PCV2 and PRRSV co-infection, and will hopefully design more targeted therapies.

## Figures and Tables

**Table 1:** Trial design (groups and infection orders of PRRSV and PCV2).

Groups	The Infection Orders of PRRSV and PCV2
Mock	no infected virus
PCV2	infection with PCV2 alone
PRRSV	infection with PRRSV alone
PCV2–PRRSV	infection with PCV2 prior to PRRSV
PRRSV–PCV2	infection with PRRSV prior to PCV2
PCV2 + PRRSV	PCV2 and PRRSV co-infection

**Table 2:** Primer sequences of the reference genes.

Genes	Primer Sequence (5’–3’)
PCV2	F 5’-CCTCACCTATGACCCCTAT-3’
	R 5’TGTTGTTTGGTTGGAAGTA-3’
PRRSV	F 5’-AAACCAGTCCAGAGGCAAGG-3’
	R 5’GCAAACTAAACTCCACAGTGTAA-3’
IFN-α	F 5’-GGATCAGCAGCTCAGGG-3’
	R 5’GAGGGTGAGTCTGTGGAAGTA-3’
IFN-γ	F 5’-AAAGATAACCAGCCCATTC-3’
	R 5’-GTCATTCAGTTTCCCAGA-3’
TNF-α	F 5’-TGGTGGTGCCGACAGATGG-3’
	R 5’-GGCTGATGGTGTGAGTGAGGAA-3’
IL-1β	F 5’-ATGCTGAAGGCTCTCCACCTC-3’
	R 5’-TTGTTGCTATCATCTCCTTGCAC-3’
IL-10	F 5’-GCATCCACTTCCCAACCA-3’
	R 5’GCAACAAGTCGCCCATCT-3’
TGF-β	F 5’-CTTACTGAGCATCTTGGACCTTA-3’
	R 5’CCACTGAGCCACAATGGAAA-3’
PD-1	F 5’-AGCCCAAGCACTTCATCCTC-3’
	R 5’-TGTGGAAGTCTCGTCCGTTG-3’
LAG-3	F 5’-CTCCTCCTGCTCCTTTTGGTT-3’
	R 5’-CAGCTCCCCAGTCTTGCTCT-3’
CTLA-4	F 5’-TCTTCATCCCTGTCTTCTCCAAA-3’
	R 5’-GCAGACCCATACTCACACACAAA-3’
TIM-3	F 5’-TTCGACGGGAGCAGTAAAGC-3’
	R 5’-AGGGCAGGACACAGTCAAAG-3’
β-actin	F 5’-CGGGACATCAAGGAGAAGC-3’
	R 5’-CTCGTTGCCGATGGTGATG-3’

## Data Availability

The data that support the findings of this study are available from the corresponding author upon reasonable request.
